# Origin of Increased Solvent Accessibility of Peptide Bonds in Mutual Synergetic Folding Proteins

**DOI:** 10.3390/ijms222413404

**Published:** 2021-12-14

**Authors:** Csaba Magyar, Anikó Mentes, Miklós Cserző, István Simon

**Affiliations:** 1 Institute of Enzymology, Research Centre for Natural Sciences, Eötvös Loránd Research Network, 1117 Budapest, Hungary; magyar.csaba@ttk.hu (C.M.); meaqaat@gmail.com (A.M.); cserzo.miklos@med.semmelweis-univ.hu (M.C.); 2 Department of Physiology, Faculty of Medicine, Semmelweis University, 1094 Budapest, Hungary

**Keywords:** intrinsically disordered proteins, mutual synergetic folding, solvent accessibility of peptide bonds, inter-subunit interaction, solvent-accessible surface area, Shannon information entropy, amino acid composition

## Abstract

Mutual Synergetic Folding (MSF) proteins belong to a recently discovered class of proteins. These proteins are disordered in their monomeric but ordered in their oligomeric forms. Their amino acid composition is more similar to globular proteins than to disordered ones. Our preceding work shed light on important structural aspects of the structural organization of these proteins, but the background of this behavior is still unknown. We suggest that solvent accessibility is an important factor, especially solvent accessibility of the peptide bonds can be accounted for this phenomenon. The side chains of the amino acids which form a peptide bond have a high local contribution to the shielding of the peptide bond from the solvent. During the oligomerization step, other non-local residues contribute to the shielding. We investigated these local and non-local effects of shielding based on Shannon information entropy calculations. We found that MSF and globular homodimeric proteins have different local contributions resulting from different amino acid pair frequencies. Their non-local distribution is also different because of distinctive inter-subunit contacts.

## 1. Introduction

The class of Mutual Synergetic Folding (MSF) proteins is a relatively newly discovered distinct class of oligomeric proteins, where a single polypeptide chain of an MSF protein is disordered, but all chains become ordered upon oligomerization [1,2]. In the case of traditional disordered proteins, there is a need for an already stable template structure. MSF proteins can fold into a stable structure without the presence of an already folded template, folding happens simultaneously with the association of the previously disordered subunits.

At the time of the discovery of the 3D structure of transmembrane proteins [3], and the “coupled folding and binding” mechanisms of disordered proteins [4,5], our knowledge of determinants of proteins structure has been expanded. The discovery of these new types of proteins was accompanied by a change of the perceivable average amino acid composition of proteins. However, the amino acid composition of MSF proteins is similar to that of globular proteins [6,7,8], which makes it difficult to identify them based on their primary structure. The different amino acid composition of disordered proteins leads to different energies, which causes the protein to have a disordered structure. A good example for the estimation of this energy is the use of statistical pairwise potentials as implemented in the IUPred prediction method, which reached recently its 3rd iteration [9]. Next to the energy, configurational entropy can also be important in disorder-to-order transitions, as shown by Liu et al. [10]. MSF proteins are special among disordered proteins because their oligomeric structure can be solved by traditional structure determination methods. In a recent publication [11] experimentally determined long intrinsically disordered protein regions were examined. The authors found that long disordered regions, which are present in MSF proteins, cannot be accurately predicted.

The tertiary structures of experimentally validated MSF proteins have been collected to the Mutual Folding Induced by Binding (MFIB) database. This database contains 205 MSF protein structures [2]. Our recent analyses [6,7] of this database aimed to find common properties among MSF proteins, which distinguish them from globular oligomeric proteins. We found that the most prominent change between MSF and globular proteins can be found in the change of solvent accessibility during the oligomerization step. The question arose as to what determines the hydration of these peptide bonds.

Sequence and structural studies of protein interactions have revealed that sequential and spatial neighboring residues often play important roles in the environmental hydrophobicity and long-term binding site interactions, thus determining the structural and functional behavior of proteins [12,13,14]. Based on this fact, the shielding effect (reducing hydration of peptide group) can be divided into local and non-local terms. The local contribution is provided by the side-chain atoms of the amino acid residues, which are connected by the peptide bond [15], while the non-local contribution is provided by the shielding effect of other sequentially distant residues [16].

In this work, we studied the solvent accessibility of peptide bonds in MSF and globular homodimeric proteins in light of relationship between dipeptide frequencies, and the ordered/disordered nature of the monomeric protein forms. Furthermore, we compared Shannon information entropy calculated from frequencies of local and spatial neighboring residues in MSF and globular proteins, which may indicate sequential differences between the two groups of proteins.

## 2. Results

**Figure 1:**
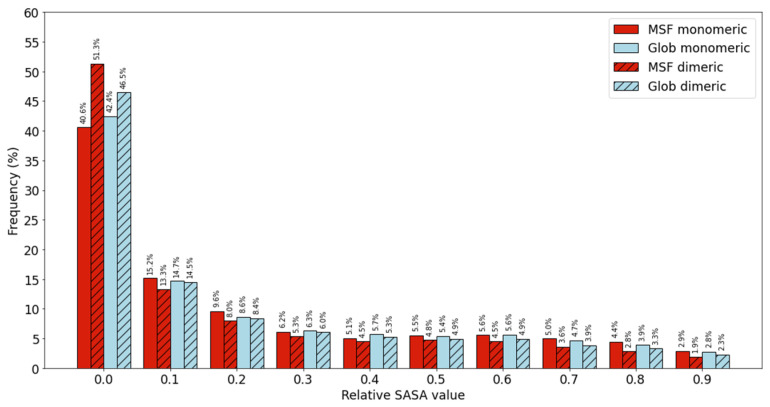
Occurrences of peptide bonds with different solvent accessibilities.

We calculated the relative solvent accessible surface area (SASA) values for all peptide bonds in our MSF and globular homodimeric (MHOD and GHOD) protein datasets for both monomeric and dimeric forms using the FreeSASA program [17]. The monomeric form was modeled by taking only a single chain of the ordered dimeric structure into account. The distribution of peptide bonds with different relative SASA values can be seen in Figure 1 for the monomeric and dimeric forms. The results are presented as histograms, where the height of a bar denotes the ratio of entries with a property in a given range. For example, the height of the first red bars refers to the percentage of peptide bonds with a relative SASA value in the [0, 0.1] interval among MSF proteins in monomeric form. There is a clear tendency in homodimeric MSF proteins to have a lower percentage of highly buried peptide bonds with lower than 10% relative SASA values in monomeric form but a higher percentage in dimeric form, when compared to globular homodimers.

**Figure 2:**
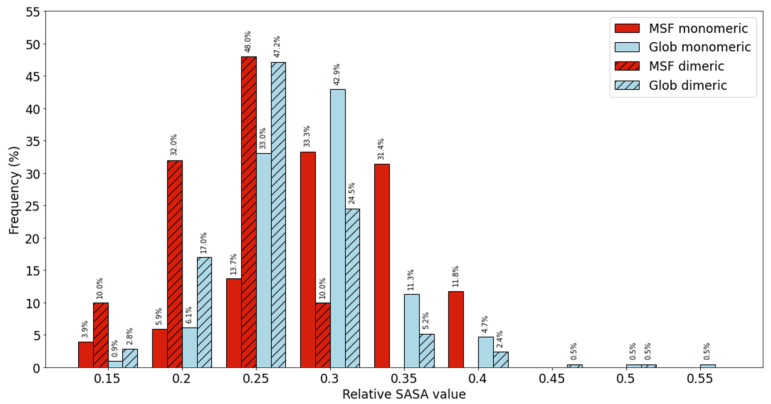
Distribution of individual proteins with different average peptide bond solvent accessibilities.

A higher number of peptides bonds become buried during dimerization in the case of MSF homodimers, than in the case of globular homodimers. Next, we calculated the solvent accessibility of peptide bonds averaged within individual proteins. We calculated the ratio of the SASA values summed over all peptide bonds divided with the sum of the reference values, thus representing the average solvent accessibility of peptide bonds within a protein. Results are presented in Figure 2 for both monomeric and dimeric calculations. The distribution of MSF average peptide bond accessibility is shifted towards higher values in the case of monomeric form, but towards lower values in dimeric form, when compared to globular proteins. These results underline our hypothesis about the importance of peptide bond solvent accessibility for MSF proteins.

**Figure 3:**
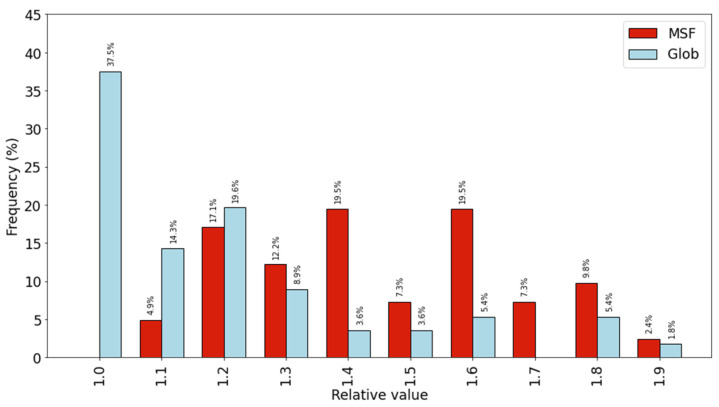
Distribution of individual proteins according to the increase of the buried/accessible peptide bond ratio.

We created a measure of the increase of peptide bond shielding upon dimerization using the following protocol. A peptide bond was identified as accessible, that is not properly shielded if its relative solvent accessibility was larger or equal than a threshold value of 10%. It was identified as buried if its relative SASA value was below 10%. We found that there are 2.6 times more not properly shielded peptide bonds in monomeric MSF proteins, than in globular proteins, which become buried upon dimerization. The number of buried (B) and accessible (A) residues were counted and we calculated the B/A ratio for both monomeric and dimeric forms. Then we calculated the dimeric B/A over the monomeric B/A quotient. This value represents the increase of peptide bond burial upon dimerization. A value of 1 would indicate that the buried/accessible residue count ratio is the same in both dimeric and monomeric forms, a value of 2 means that in the dimeric form the buried/accessible ratio is twice the monomeric value. Results can be seen in Figure 3. The distribution of the MSF protein values is shifted toward higher values, meaning that in the case of MSF proteins the ratio of the buried/accessible residues is higher in the dimeric form.

**Figure 4:**
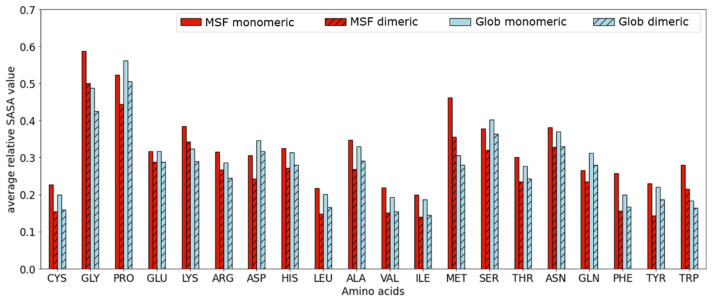
Average relative main-chain SASA values according to residue types.

These results implicate, that solvent accessibility of the peptide bonds is an important factor in the destabilization of the monomeric form of MSF proteins. We investigated if amino acid composition plays a role in the above-presented findings. Instead of the peptide bond relative SASA value, which involves two amino acids, the relative main-chain SASA values were compared for the 20 residue types. The data presented in Figure 4 shows increased average SASA values for the glycine, lysine, methionine, and tryptophan residues in MSF proteins, while slightly decreased values for proline, aspartic acid, serine and glutamine residues. However, most values are rather similar in the MHOD and the GHOD protein datasets.

Glycine and proline residues have the highest average main-chain accessibility in both MSF and globular proteins, while cysteine, leucine, valine, and isoleucine residues have the lowest average values in both datasets. Traditional disordered proteins can be distinguished from globular ones based on their amino acid composition. Because of the similar amino acid composition of MSF and globular proteins [6,7,8], statistical measures based on the residue composition did not help to separate the two types of proteins, thus we decided to compare their Shannon information entropy content [18]. The probability distribution of the individual amino acids was calculated as their observable frequency using Equation (1).

Equation (1): Calculation of the frequencies of the *i*th amino acid type

(1) $$P_{i}=\frac{N_{i}}{N_{tot}}\;,$$

where *N_i_* is the number of the *i*th amino acid type and *N_tot_* is the total number of amino acids in the actual dataset.

For calculating entropy values of individual proteins, we decided to use a normalized version of the relative Shannon information entropy, frequently referred to as the Kullback–Leibler divergence [19] using Equation (2). It measures how our *P_i_* amino acid probability distribution differs from a reference *Q_i_* probability distribution. We are using reference *Q_i_* values obtained from a non-redundant subset of the PDB database [20] (see PDB codes in Table S1A), which is significantly larger than our homodimeric protein datasets. The $P_{i}log\frac{P_{i}}{Q_{i}}$ values obtained for the 20 amino acids are listed in Table S2A,B, calculated from the amino acid compositions of the GHOD and MHOD protein datasets, respectively. Entropy values for individual proteins were calculated using Equation (2), where entropy values were normalized using a division with log N to avoid size dependence of the values.

Equation (2): Calculation of the normalized Shannon information entropy for an individual protein based on the amino acid composition

(2) $$S\left(j\right)=\frac{1}{\log N_{j}}\sum_{i=1}^{N_{j}}P_{i}log\frac{P_{i}}{Q_{i}}\;,$$

where *N_j_* is the number of amino acids in protein *j.*

**Figure 5:**
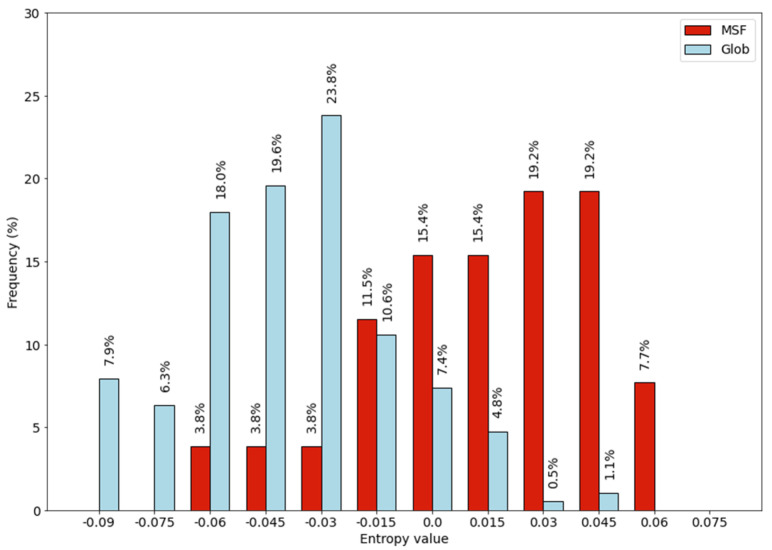
Entropy values calculated based on the amino acid composition.

Entropy values were calculated for entries in our homodimeric protein datasets. Results are presented as a histogram in Figure 5.

There is a difference between the distributions obtained on MSF and globular homodimers, entropy values of the MSF proteins are shifted toward positive values. This result is somewhat unexpected in light of our previous results. Recently [6] we compared the amino acid composition of MSF and globular homodimers using principal component analysis and we found no significant difference. The Shannon entropy calculation seems to be more sensitive than our previous analysis.

As we have found a significant difference in the burial of peptide bonds between MSF and globular homodimers, we started to look for a possible reason. Since local shielding of the peptide bonds is mainly provided by the two amino acids creating the peptide bond, we calculated the dipeptide frequencies in our MHOD and GHOD protein datasets by dividing the count number of a specific dipeptide with the total number of peptide bonds using Equation (3).

Equation (3): Calculation of the frequencies of the *ij* dipeptides

(3) $$P_{ij}=\frac{N_{ij}}{N_{pb}},$$

where *N_pb_* is the total number of peptide bonds.

**Figure 6:**
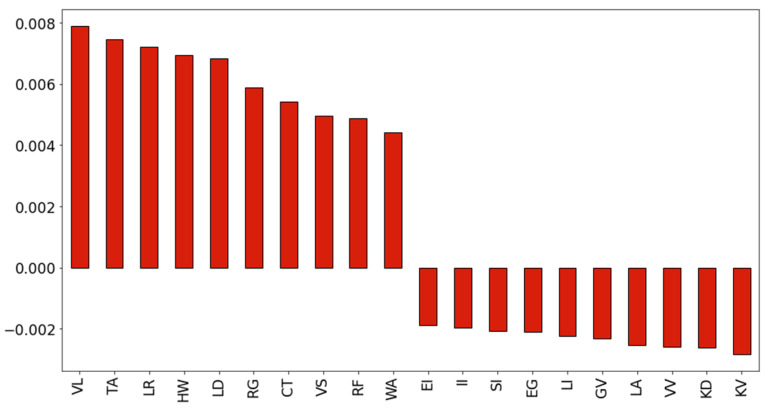
Dipeptides with the highest and lowest entropy-like contributions.

To pinpoint the differences, we calculated relative entropy-like $P_{ij}log\frac{P_{ij}}{Q_{ij}}$ values for all dipeptides using *P_ij_* values obtained on the MHOD protein dataset, and reference *Q_ij_* values obtained on the GHOD protein dataset. The highest and lowest 10 values are plotted in Figure 6.

There are only a handful of dipeptides that have a strong preference for MSF or globular proteins, but most dipeptides have rather weak preference values. Though the amino acid composition of MSF homodimeric proteins is close to that of globular proteins [6,8], differences can be found in their sequence already at the dipeptide level.

Based on this observation dipeptide distribution might discriminate MSF proteins from globular ones, so we investigated the information content of the protein sequences. We calculated Shannon information entropy values based on dipeptide frequencies, similar to the previous case of amino acid compositions. The $P_{ij}log\frac{P_{ij}}{Q_{ij}}$ values calculated using our non-redundant sequence dataset derived *Q_ij_* and *P_ij_* values obtained on the GHOD and MHOD protein datasets can be found in Table S3A,B, respectively. Entropy values for individual proteins were calculated using Equation (4).

Equation (4): Calculation of the Shannon information entropy for an individual protein based on the dipeptide frequencies:

(4) $$S\left(k\right)=\frac{1}{\log N_{k}}\sum_{1}^{N_{k}}P_{ij}log\frac{P_{ij}}{Q_{ij}}\;,$$

where *N_k_* is the number of peptide bonds in protein *k.*

**Figure 7:**
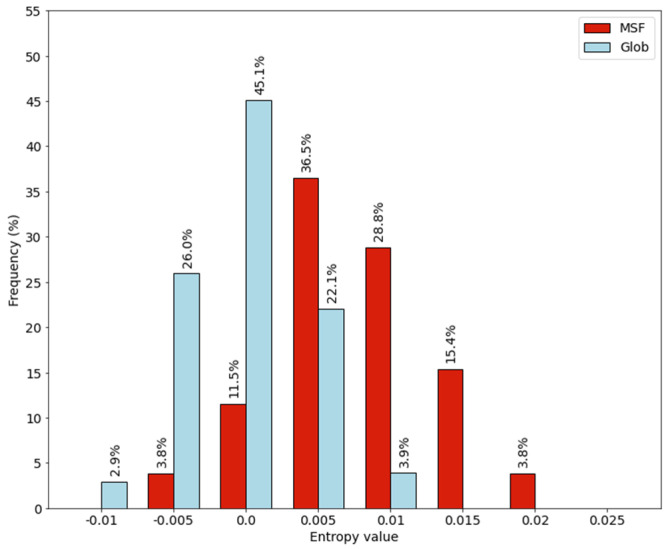
Entropy values calculated from the dipeptide frequencies.

We plotted the entropy value distribution of individual proteins as histograms (see Figure 7). We can see a similar effect as in the case of entropy values calculated from the amino acid compositions. There is a shift in the distribution of MSF proteins towards positive values.

To investigate the effect of the non-local long-range shielding of peptide bonds upon dimerization we created the following protocol. We identified inter-subunit residue contacts based on a simple distance criterion. Residues pairs were identified as important for long range shielding, if the participating residues are part of different protein chains and they have at least one heavy-atom pair with a distance shorter than 4 Ångströms. We identified all these residue pairs and calculated their frequencies using Equation (5).

Equation (5): Calculation of the frequencies of the *ij* residue pairs

(5) $$P_{ij}=\frac{N_{ij}}{N_{tc}},$$

where *N_tc_* is the total number of contacting residue pairs.

**Figure 8:**
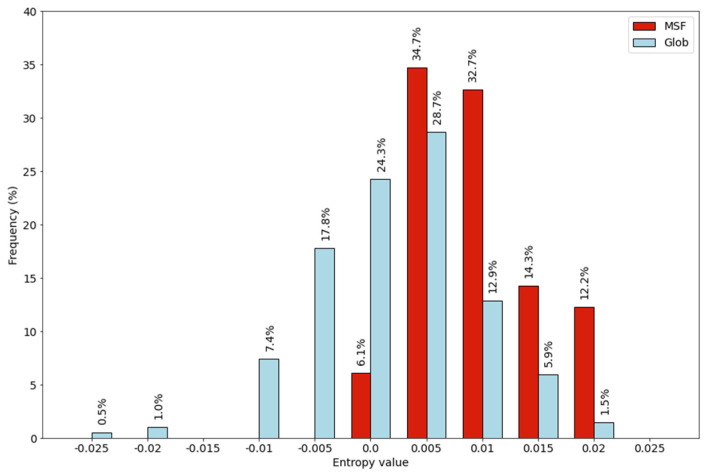
Entropy values calculated from the inter-subunit contacts.

Since reference *Q_ij_* values can be derived only from dimeric structures, the GHOD protein dataset was used for this purpose. In order to produce entropy values also for globular proteins, both MHOD and GHOD protein datasets were scored using the MHOD derived *P_ij_* values. The $P_{ij}log\frac{P_{ij}}{Q_{ij}}$ values for all residue pairs can be found in Table S4. Relative entropy values for individual proteins were calculated similarly to the previous cases, but the sum was created over all contacting residue pairs using Equation (6). The distribution of these values can be seen in Figure 8.

Equation (6): Calculation of the Shannon information entropy for an individual protein based on inter-subunit contacts:

(6) $$S\left(k\right)=\frac{1}{\log N_{c}}\sum_{1}^{N_{c}}P_{ij}log\frac{P_{ij}}{Q_{ij}},$$

where *N_c_* is the number of contacts within protein *k.*

Despite the same *P_ij_* matrix was used for both datasets, there is a shift in the distribution of the MSF protein values towards positive values. There is an overlap of the two distributions, but more than a quarter of the globular proteins have negative values, while all MSF proteins have positive values. About one fifth of the globular proteins has a value larger than 0.01, while more than half of the MSF proteins are found in this range.

## 3. Discussion

MSF proteins are a relatively new class of proteins with little knowledge about their folding. Our current comparison of MSF and globular homodimeric proteins provided the following results. MSF proteins have a higher average relative solvent accessibility of the peptide bonds in their monomeric form. Upon dimerization, a higher proportion of accessible peptide bonds become buried in the case of MSF homodimers, when compared to globular ones. A significant increase in the number of both buried peptide bonds and buried residues upon oligomerization is characteristic for MSF proteins. Zhou et al. recently analyzed the normalized monomer surface area versus normalized interfacial surface area in a recent publication [21]. Their findings are in agreement with ours about the relevance of the increased inter-subunit surface area in MSF proteins.

The burial of the peptide bonds from solvent molecules is established by shielding through local and non-local residues, relative to the actual peptide bond. We found differences in both local and non-local dipeptide frequencies between MSF and globular homodimers using Shannon information entropy calculations. This behavior originates from the different dipeptide frequencies locally and different inter-subunit contacts non-locally.

Zhou et al. also emphasized the importance of intrinsic disorder in complex formation. Previously we found [6] that on our filtered homodimeric protein dataset, all seven tested methods predicted less than 30% of the residues as disordered, while the average value was around 14%. Our suggestion is that a simple physicho-chemical property may be responsible for the destabilization of MSF monomers. Our results indicate that despite the similar amino acid compositions [6], MSF and globular homodimeric proteins have different amino acid pair statistics, leading to different Shannon information entropy distributions. We suggest that this change in the dipeptide frequencies can be accounted for by the less efficient shielding of the peptide bonds of MSF proteins in their monomeric forms. This phenomenon can provide an important contribution to the destabilization of monomeric MSF protein chains, by disturbing the hydrogen bond network of the protein backbone, leading to the disruption of secondary structural elements. The different non-local entropy values may result from the increased necessity of proper peptide bond shielding during the dimerization step.

## 4. Materials and Methods

For the database analyses, we wrote our own Python programs using Biopython extensions [22] and created a sequence and two structural datasets of proteins with known three-dimensional structures. First, we created a larger sized non-redundant sequence dataset of PDB entries with less than 40% sequence identity using the Mufold-DB database [23] for reference purposes. PDB codes with the protein chain designation can be found in Table S1A. This low similarity cutoff value ensures that the entries are not too similar, which could have biased our residue pair statistics. We used a structural database as a starting point on purpose to include sequences of globular proteins. The resulting dataset contains around 23,000 entries, which is already large enough to provide reliable statistics. We created the MHOD protein dataset by collecting homodimeric MSF proteins found in the MFIB database [2]. We created as reference the GHOD protein dataset of globular homodimeric proteins based on the non-redundant PDB-Filter select 2017 database [24]. Since our aim is to understand which features differentiate MSF proteins from globular ones, we applied a volume/surface criterion as described in our previous publication [7]. This filtering step retains only compact globular like structures and eliminates rather one dimensional, rod-like proteins (like collagen), which would have biased our solvent accessibility calculations. Protein surfaces and volumes were calculated using the FreeSASA 2.03 [17] and the ProteinVolume 1.3 programs [25], respectively. SASA values were calculated for both dimeric and monomeric forms of all proteins. The monomeric form was modeled by deleting the second protein chain from the PDB files. During SASA calculation a couple of additional structures were excluded from the datasets because of structural problems influencing our calculations. A typical problem was that the different protein chains were not in contact, thus SASA values calculated from the dimeric and the monomeric forms were almost identical. The final list of the PDB codes of the remaining 52 entries, called the MHOD protein dataset, can be found is Table S1B. To match the size distribution of this dataset, only proteins with less than 300 residues were kept in the GHOD protein dataset. The list of the PDB codes of the remaining 203 GHOD proteins can be found in Table S1C.

In our previous publications, the solvent accessibility of the main chain was handled at the residue level [6,7]. In this work we focus on the solvent accessibility of peptide bonds, thus SASA values were calculated for atoms forming the peptide bonds. We utilized the absolute all-atom SASA values. The absolute SASA value of a peptide bond was the sum of the atomic absolute SASA values (N, CA, C and O) belonging to a peptide bond. To characterize the relative accessibility of peptide bonds, we created reference SASA values (see Table S5) for all 400 Ala-X-Y-Ala tetrapeptides, built in extended conformation using PyMOL [26]. The relative solvent accessibility of a peptide bond was calculated by dividing its absolute SASA value with the appropriate reference value. We calculated the relative SASA values of peptide bonds of all MHOD and GHOD entries in both their monomeric and dimeric forms.

When available, modified PDB files were used from the MFIB database. In the case of NMR structures, the representative model structure was selected based on the OLDERADO NMR resource found in PDBe [27].

## Data Availability

Data supporting the reported results can be found in the Supplement.

## Supplementary Materials

The following are available online at https://www.mdpi.com/article/10.3390/ijms222413404/s1.

## Abbreviations

GHOD: Globular HomoDimeric; IDP: Intrinsically Disordered Protein; MFIB: Mutual Folding Induced by Binding; MHOD: MSF HomoDimeric; NMR: Nucleic Magnetic Resonance; MSF: Mutual Synergetic Folding; OLDERADO: On Line Database of Ensemble Representatives And Domains; PDB: Protein Data Bank; SASA: Solvent Accessible Surface Area.
